# An Assessment of the Utility of Serum Fructosamine in the Diagnosis and Monitoring of Diabetes Mellitus

**DOI:** 10.7759/cureus.33549

**Published:** 2023-01-09

**Authors:** Jyoti John, Apurva Sakarde, Janhvi Chafle, Dnyanesh Amle, Jancy Jose, Vibha Sakhare, Bharatsing D Rathod

**Affiliations:** 1 Biochemistry, All India Institute of Medical Sciences, Nagpur, IND; 2 General Internal Medicine, All India Institute of Medical Sciences, Nagpur, IND

**Keywords:** glycated haemoglobin, diabetes mellitus, glycated albumin, hba1c, protein corrected fructosamine, albumin corrected fructosamine, fructosamine

## Abstract

Background: Fructosamine (FA) has gained importance as a new biomarker for hyperglycemia in the past decade and may be of indispensable use in certain conditions where hemoglobin A1c (HbA1c) falls short of utility such as disorders of red blood cells, patients with rapid glycemic excursions requiring more short-term monitoring, pregnancy, chronic kidney disease, etc.

Methods: The present study was a hospital-based observational cross-sectional study conducted in the Department of Biochemistry, All India Institute of Medical Sciences (AIIMS), Nagpur, India. Serum HbA1c, FA, albumin-corrected fructosamine (AlbF), total protein-corrected FA (PrF), hemoglobin (Hb), and hematocrit (Hct) were estimated in 32 controls (Group I) and 32 cases of diabetes mellitus (DM) (Group II). The clinical data and lab results were presented as mean±SD/±standard error (SE) of the mean. Student’s t-test and ANOVA were used to compare various parameters between the groups. Pearson correlation analysis was performed to assess the correlation between different diagnostic parameters. The receiver operating characteristic (ROC) curve was plotted to assess the diagnostic significance and cut-off value for FA, AlbF, and PrF.

Results: The controls and cases were matched for age and gender distribution. Serum HbA1c (p<0.0001), serum FA (p<0.0001), fasting blood sugar (p=0.001), postprandial blood sugar (p<0.0001), random blood sugar (p=0.001), hematocrit (p=0.002), AlbF (p<0.0001), and PrF (p<0.0001) were found to be significantly higher in known diabetic subjects compared to controls. The case group was further subdivided into pre-diabetic and diabetic groups. On correlation analysis of HbA1c with various parameters, a moderate correlation of HbA1c was noted with FA (r=0.522, p<0.0001) and AlbF (r=0.375, p=0.002) in all subjects. Additionally, a moderate correlation of FA (r=0.479, p=0.033), AlbF (r=0.444, p=0.050), and PrF (r=0.441, p=0.065) with HbA1c was also found in subjects with diabetic range glycemia. No such correlation was noted in the pre-diabetic group. No significant correlation was noted between FA and its corrected values in any range of glycemia. None of the parameters assessing glycemia were found to be significantly affected by hemoglobin status. On ROC curve analysis, HbA1c was found to be the best parameter (area under the curve (AUC) =83%, p<0.0001) followed by AlbF (AUC= 80.5%, p<0.0001) and uncorrected FA (AUC=80.5%, p<0.0001) to diagnose DM.

Conclusion: Serum FA should be considered a valid diagnostic biomarker and of indispensable use in special populations where HbA1c falls short of utility such as patients with red blood cell disorders or those showing rapid glycemic excursions such as those on corticosteroid therapy or insulin therapy, etc. It exhibits additional advantages over HbA1c with respect to lower reagent cost and easy automation on any conventional laboratory instruments based on simple colorimetry.

## Introduction

Diabetes mellitus (DM) is a metabolic disorder characterized by increased blood glucose levels resulting from either defective insulin secretion, insulin activity, or both [1]. Globally, one in every 11 adults is reported to be suffering from DM, out of which 90% belong to the type 2 DM category [2]. According to Wild et al., it is predicted that the prevalence of DM in India is expected to rise from 40.6 million in 2006 to 79.4 million by 2030 [3]. By 2035, an estimated 592 million individuals are predicted to die of diabetes globally [4]. The condition is a serious global health burden not only because of its prevalence but also because of its frequent and life-threatening complications such as cardiovascular disease (CVD), diabetic nephropathy, retinopathy, etc. The insidious onset and long duration of asymptomatic disease before the development of symptoms predispose the patient to be associated with complications at the time of diagnosis itself [5]. Thus, it is imperative to search for investigations that can be helpful in screening, early diagnosis, and effective monitoring of glycaemic control.

Currently, the diagnostic tests utilized for DM are plasma glucose and HbA1c levels as per the American Diabetes Association (ADA) criteria [1,6]. Glycated hemoglobin or hemoglobin A1c (HbA1c) is used as an important diagnostic indicator of long-term glycaemic control and the investigation of choice for treatment monitoring in diabetics. However, HbA1c levels are in turn dependent on the longevity of red blood corpuscles (RBC). For example, in patients with anemic states falsely increased HbA1c values are seen, whereas treatment brings in a spurious decrease [7]. Hemolytic disorders such as sickle hemoglobin (HbS) and hemoglobin C (HbC) cause decreased lifespan of RBCs and thus render HbA1c unreliable for monitoring [8]. Some subjects are genetically predisposed to have hyperglycation [9]. Fructosamine (FA), which is a measure of non-enzymatic glycation of circulating proteins including albumin, globulins, and lipoproteins, appears to be a reasonable alternative to HbA1c measurement in situations where HbA1c is not reliable. 

In the present study, we aimed to estimate serum FA levels, albumin-corrected fructosamine (AlbF) levels, and total protein-corrected fructosamine levels (PrF) in cases of DM and healthy controls and compare the values of both groups. We also attempted to study the correlation of HbA1c levels with FA levels, AlbF, and PrF levels in the subjects, to assess their efficacy in indicating glycemic control. The cut-off values for these parameters were also derived.

## Materials and methods

The present study was a hospital-based observational cross-sectional study conducted in the Department of Biochemistry, All India Institute of Medical Sciences (over one month in November 2022. All study protocols adhered to the Declaration of Helsinki. Permission of the Institutional Ethical Committee was sought prior to the study (approval no.: AIIMS-NAG/IEC/Pharmac/2022/473). Thirty-two pre-diagnosed cases of DM along with 32 age and gender-matched controls were recruited for the study. 

The controls group (Group I) consisted of normoglycemic healthy volunteers in the age group of 30 to 75 years without any apparent ongoing acute or chronic inflammatory condition who came to AIIMS, Nagpur for routine medical checkups, medical fitness, or were accompanying patients visiting the out-patient department (OPD). The cases group (Group II) consisted of type 2 DM patients in the age group of 30 to 75 years attending the OPD or were admitted to the inpatient department (IPD) of AIIMS, Nagpur. The diagnostic criteria to diagnose DM were as per the diagnostic criteria set by the ADA i.e., fasting plasma glucose values of ≥ 126 mg/dl, two-hour post-load plasma glucose ≥ 200 mg/dl, HbA1c ≥ 6.5%; or random blood glucose ≥ 200 mg/ dl. An HbA1c value between 5.7% to 6.4%, fasting plasma glucose of 100 to 126 mg/dL, or two-hour plasma glucose between 140 to 199 mg/dL during an oral glucose tolerance test (OGTT) indicated an increased risk of DM i.e., the pre-diabetes state [1].

Cases or controls with a history of ischemic heart disease, hypertension, vascular disease, or with any history of an acute or chronic infectious or inflammatory disease such as tuberculosis, autoimmune disorders, connective tissue disorders, etc., or with any history of lung, renal or hepatic disease was excluded from the study. Subjects with any history of drug intake that may affect blood glucose levels such as steroids, antihypertensive drugs, etc., were also excluded. Taking all sterile precautions, venous blood samples were collected from all study subjects and transferred to labeled ethylenediamine tetraacetic acid (EDTA) and plain vacutainer tubes. The EDTA samples were processed for HbA1c and other complete blood count parameters on the Adams HT8180 (Arkray Ameica Inc., Minneapolis, MN, USA)analyzer based on the high-performance liquid chromatography (HPLC) method and the Sysmex XN 1000 Haematology Analyzer (Sysmex Corp., Kobe, Japan), respectively. The serum obtained on the centrifugation of plain vacutainers at 3000 rpm for 10 minutes was separated and processed for routine parameters like blood glucose (random/fasting and post-meal), serum total protein, and albumin on the Vitros 5600 Integrated System (Ortho Clinical Diagnosis, Raritan, NJ, USA) dry chemistry analyzer. The remaining serum was aliquoted and stored at −80°C until further analysis for serum FA levels. Frozen samples were thawed to room temperature and analyzed for serum FA on Transasia XL 1000 (Transasia Bio-Medicals Ltd., Mumbai, MH, India) fully automated autoanalyzer using a colorimetric kit based on the nitro-blue tetrazolium (NBT) method. The principle of the kit is based on the fact that FA present in the blood can convert NBT dye to formazan under alkaline conditions. The quantity of formazan formed is directly proportional to the FA present in the blood, which is read photometrically at 540 nm. The FA values were corrected for serum total protein and albumin concentrations as per the following formulae [10]: serum FA corrected for total PrF = (FA/Total Protein) x 7.0 g/dl; serum FA corrected for AlbF = (FA/Albumin) x 4.1 g/dl.

Data were statistically analyzed using the Statistical Package for Social Sciences (SPSS) software, version 21 (IBM Corp., Armonk, NY, USA). The demographic and lab data were expressed as mean±SD/±SE of the mean. Kolmogorov-Smirnov analysis was done to check for the linearity of data. Student’s t-test and ANOVA were used to compare various parameters between the groups. Pearson correlation analysis was performed to assess the correlation between different diagnostic parameters. The ROC curve was plotted to assess the diagnostic significance and cut-off value. A value of p<0.05 was taken as statistically significant.

## Results

**Figure 1:**
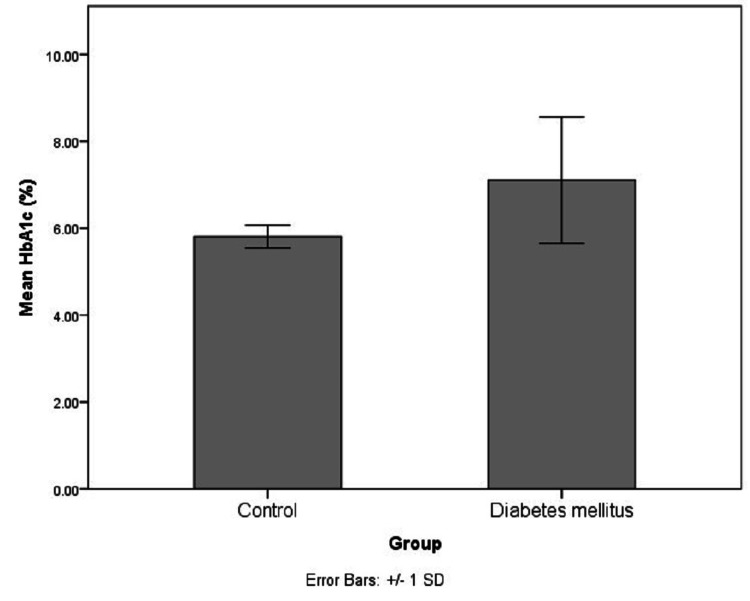
Distribution of HbA1c levels in the study groups HbA1c: Hemoglobin A1c

**Figure 2:**
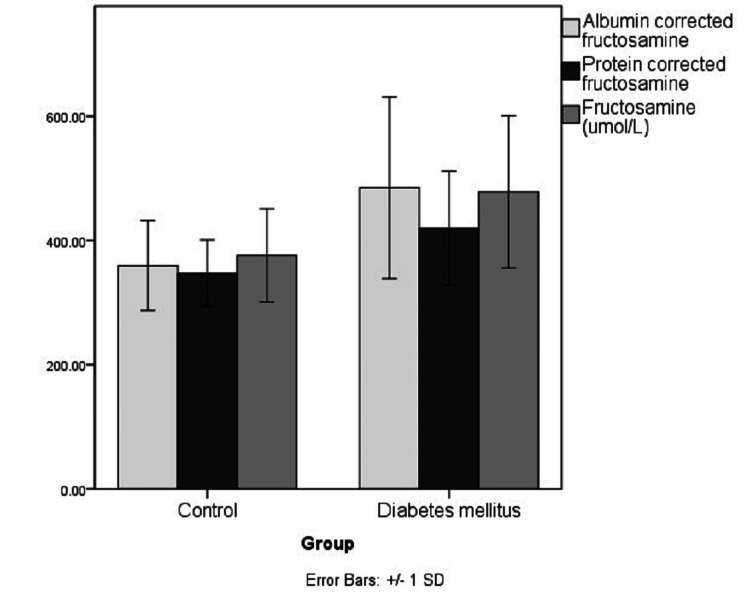
Distribution of FA, AlbF, and PrF levels in the study groups FA: Fructosamine, AlbF: Albumin-corrected fructosamine, PrF: Protein-corrected fructosamine

**Figure 3:**
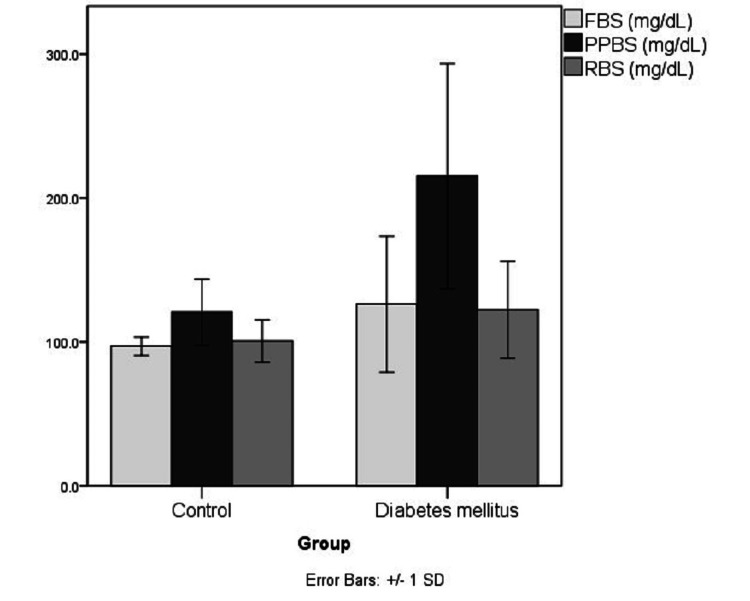
Distribution of fasting, post-meal, and random blood sugar levels in the study groups FBS: Fasting blood sugar levels, PPBS: Postprandial blood sugar levels, RBS: random blood sugar levels

**Figure 4:**
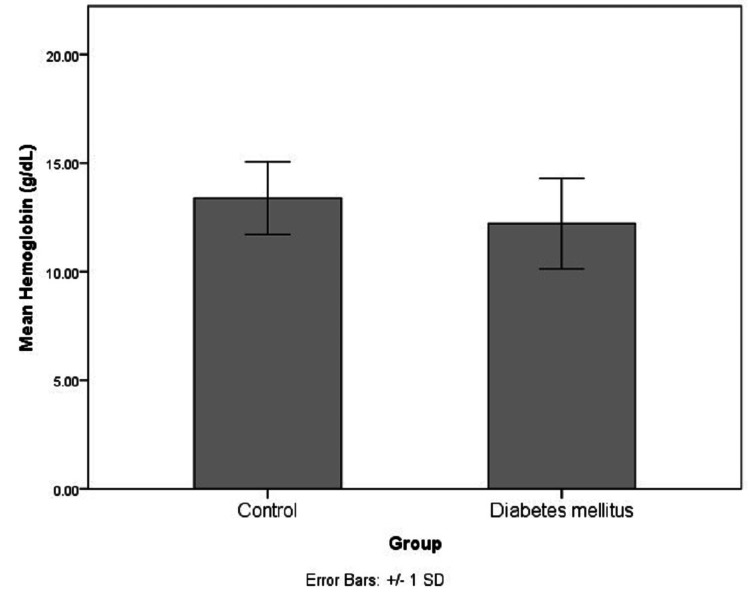
Distribution of Hb levels in the study groups Hb: Hemoglobin

**Figure 5:**
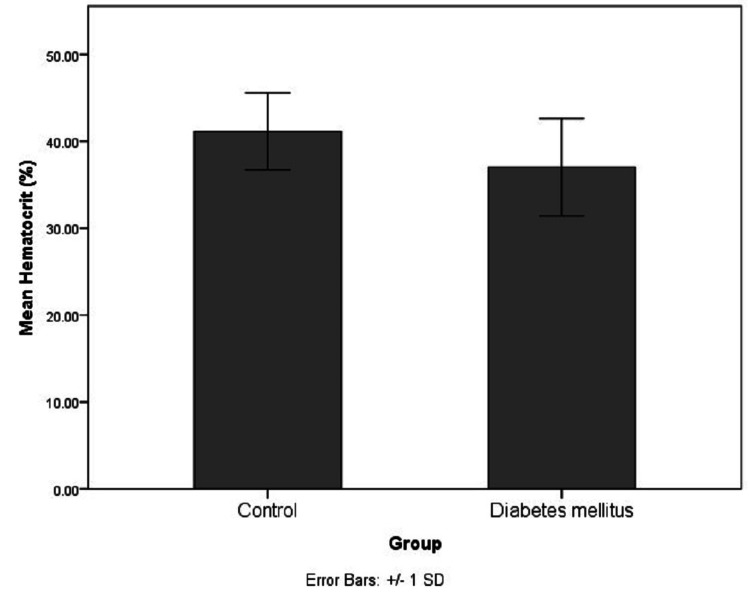
Distribution of Hct levels in the study groups Hct: Hematocrit

The demographical and biochemical characteristics of the study population are represented in Table 1 and Figures 1-5. The controls and cases were found to be matched for age (p=0.89) and gender distribution (p=0.20). The HbA1c (p<0.0001), serum FA (p<0.0001), fasting blood sugar (p=0.001), post-meal blood sugar (p<0.0001), random blood sugar (p=0.001), hematocrit (Hct) (p=0.002), AlbF (p<0.0001), and PrF (p<0.0001) were found to be significantly higher in known diabetic subjects as compared to controls. Hemoglobin (Hb) levels were found to be significantly higher in the control group (p=0.016).

**Table 1:** Comparison of demographical and biochemical characteristics of the study subjects

Parameter		Controls (N=32)	Diabetes mellitus (N=32)	T-value	p-value
Age (Years)		47.91±15.64	54.22±13.49	-1.729	0.089
Gender n (%)	F	15 (46.9%)	10 (31.2%)	1.641	0.20
	M	17 (53.1%)	22 (68.8%)		
HbA1c (%)		5.81±0.26	7.1±1.45	-4.973	<0.0001
FA (umol/L)		376.19±74.9	478.33±122.47	-4.025	<0.0001
Fasting blood sugar (mg/dL)		96.99±6.39	126.18±47.22	-3.465	0.001
Post-meal blood sugar (mg/dL)		120.7±23.12	215.12±78.22	-6.548	<0.0001
Random blood sugar (mg/dL)		100.66±14.75	122.31±33.71	-3.328	0.001
Haemoglobin (Hb) (g/dL)		13.38 ±1.67	12.21± 2.08	2.47	0.016
Hct (%)		41.13±4.44	37.02±5.6	3.259	0.002
AlbF		359.67±72.64	485.18±146.22	-4.349	<0.0001
PrF		347.49±53.46	420.03±91.8	-3.863	<0.0001

**Figure 6:**
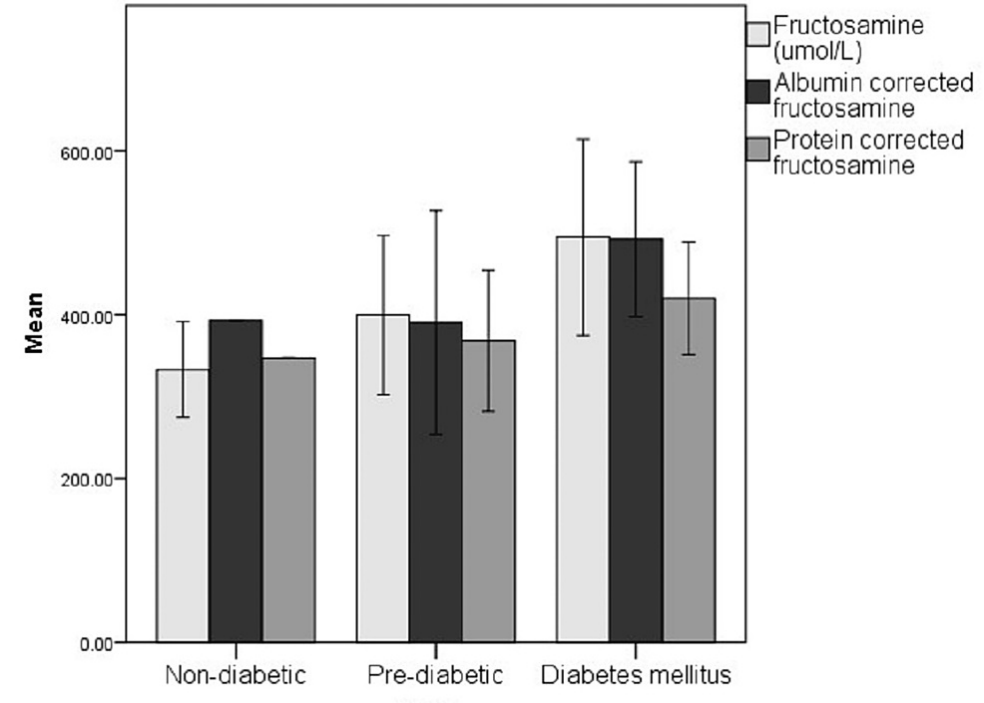
Distribution of FA, AlbF, and PrF levels in the non-diabetic, pre-diabetic and diabetic groups FA: Fructosamine, AlbF: Albumin-corrected fructosamine, PrF: Protein-corrected fructosamine

**Figure 7:**
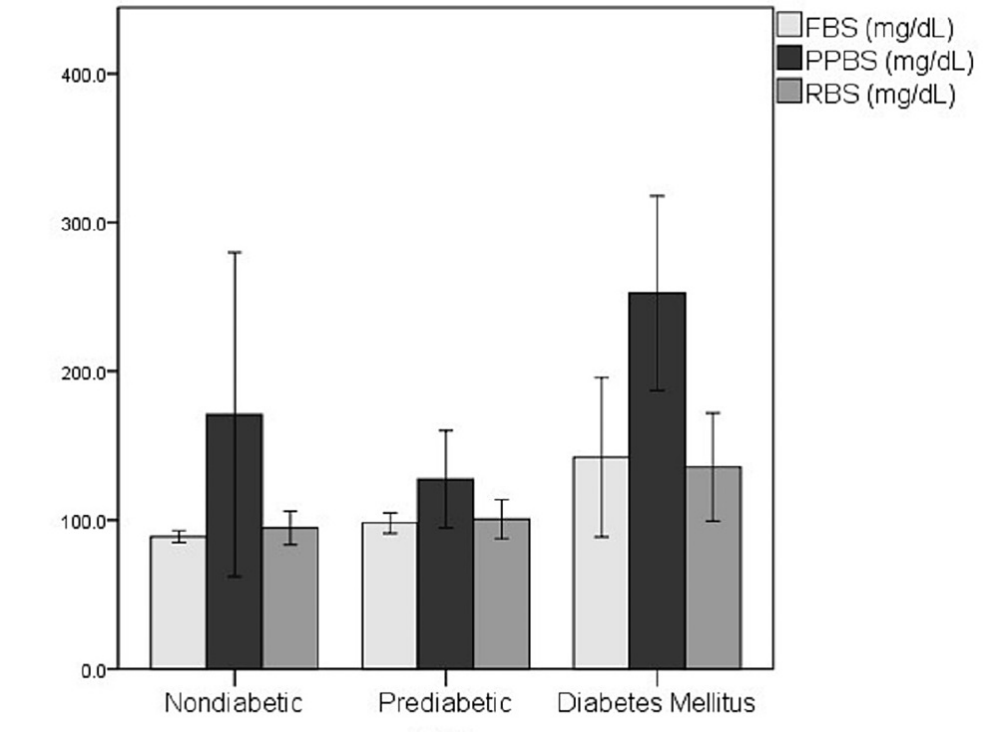
Distribution of fasting, post-meal, and random blood glucose levels in the non-diabetic, pre-diabetic, and diabetic groups FBS: Fasting blood sugar levels, PPBS: Postprandial blood sugar levels, RBS: Random blood sugar levels

**Figure 8:**
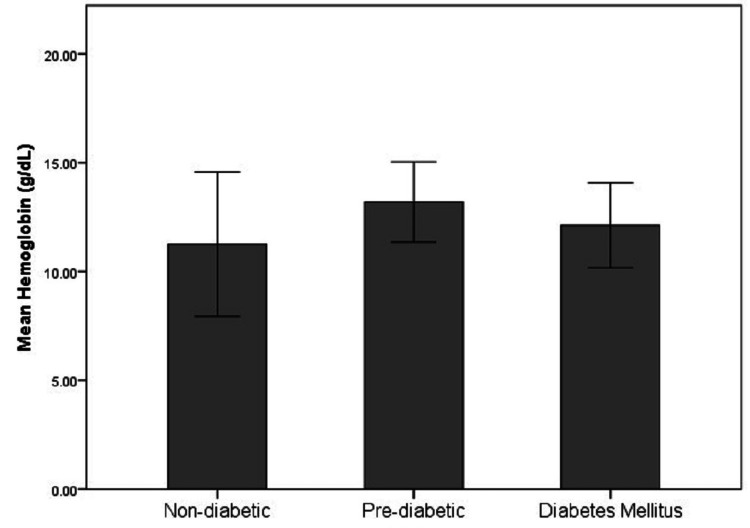
Distribution of Hb levels in the non-diabetic, pre-diabetic, and diabetic groups Hb: Hemoglobin

**Figure 9:**
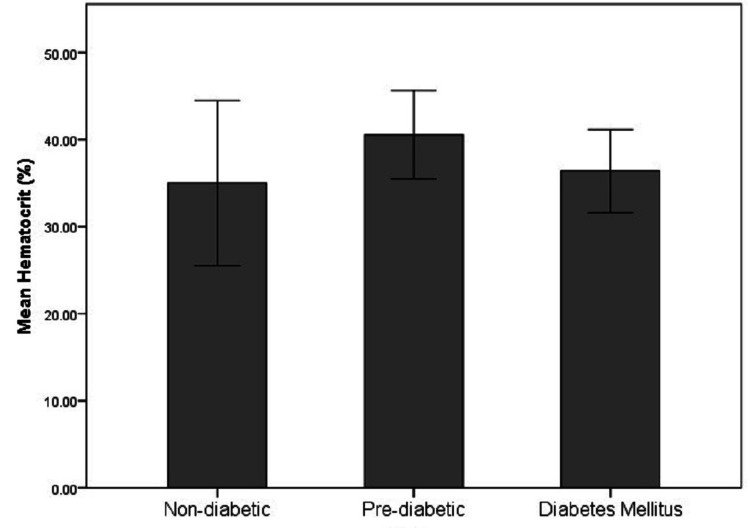
Distribution of Hct levels in the non-diabetic, pre-diabetic, and diabetic groups Hct: Hematocrit

For further analysis, the case groups were subdivided into pre-diabetic and diabetic groups based on glycemic control as per their HbA1c levels. The distribution of the glycemic index markers in each of the sub-categorized groups has been depicted in Figures 6-9.

On correlation analysis of HbA1c with various parameters, a moderate correlation of HbA1c was noted with FA (r=0.522, p<0.0001) and AlbF (r=0.375, p=0.002) in all subjects. Additionally, moderate correlation of FA (r=0.479, p=0.033), AlbF (r=0.444, p=0.050), and PrF (r=0.441, p=0.065) with HbA1c was also found. However, no such correlation was noted in the pre-diabetic group (Table 2).

**Table 2:** Correlation of HbA1c with various parameters HbA1c: Hemoglobin A1c, FA: Fructosamine, AlbF: Albumin-corrected fructosamine, PrF: Protein-corrected fructosamine, Hct: Hematocrit

Parameter	All samples (n=64)		Pre-diabetic range HbA1c		Diabetic range HbA1c	
	r	p-value	r	p-value	r	p-value
FA	0.522	<0.0001	0.257	0.101	0.479	0.033
AlbF	0.375	0.002	-0.075	0.636	0.444	0.050
PrF	0.107	0.400	-0.90	0.569	-0.421	0.065
Hct	-0.281	0.025	-0.300	0.054	0.003	0.931

No significant correlation was noted between FA and its corrected values in any range of glycemia (Table 3).

**Table 3:** Correlation of various parameters with fructosamine HbA1c: Hemoglobin A1c, AlbF: Albumin-corrected fructosamine, PrF: Protein-corrected fructosamine, Hct: Hematocrit

Parameter	All samples (n=64)		Pre-diabetic range HbA1c		Diabetic range HbA1c	
	r	p-value	r	p-value	R	p-value
AlbF	0.285	0.022	0.181	0.252	0.142	0.551
PrF	0.242	0.054	0.302	0.052	-0.222	0.347
Hct	-0.267	0.037	-0.291	0.061	0.085	0.712

Various parameters were compared between subjects with anemia, normal Hb, and higher than normal Hb. None of the parameters was found to be significantly different among these three groups (Table 4).

**Table 4:** Comparison of various parameters between subjects with different hemoglobin levels HbA1c: Hemoglobin A1c, FA: Fructosamine, AlbF: Albumin-corrected fructosamine, PrF: Protein-corrected fructosamine, Hct: Hematocrit, Hb: Hemoglobin

Parameter	Anaemic (n=21)	Normal Hb (n=35)	High Hb (n=08)	F-value	p-value
HbA1c (%)	6.78±1.2	6.36±1.31	6.01±0.62	1.390	0.257
FA (µmol/L)	440.9±121.3	421±112.56	418.68±103.26	0.224	0.800
AlbF	443.11±117.92	411.02±143.92	418.05±108.42	0.392	0.677
PrF	398.35±88.81	368.44±77.22	412.51±88.04	1.419	0.250
Hb (g/dL)	10.55±0.98	13.45±0.91	15.81±0.49	120.7	<0.0001
Hct (%)	33.42 ± 3.85	41.08 ± 3.15	45.12 ± 4.05	44.901	<0.0001

**Figure 10:**
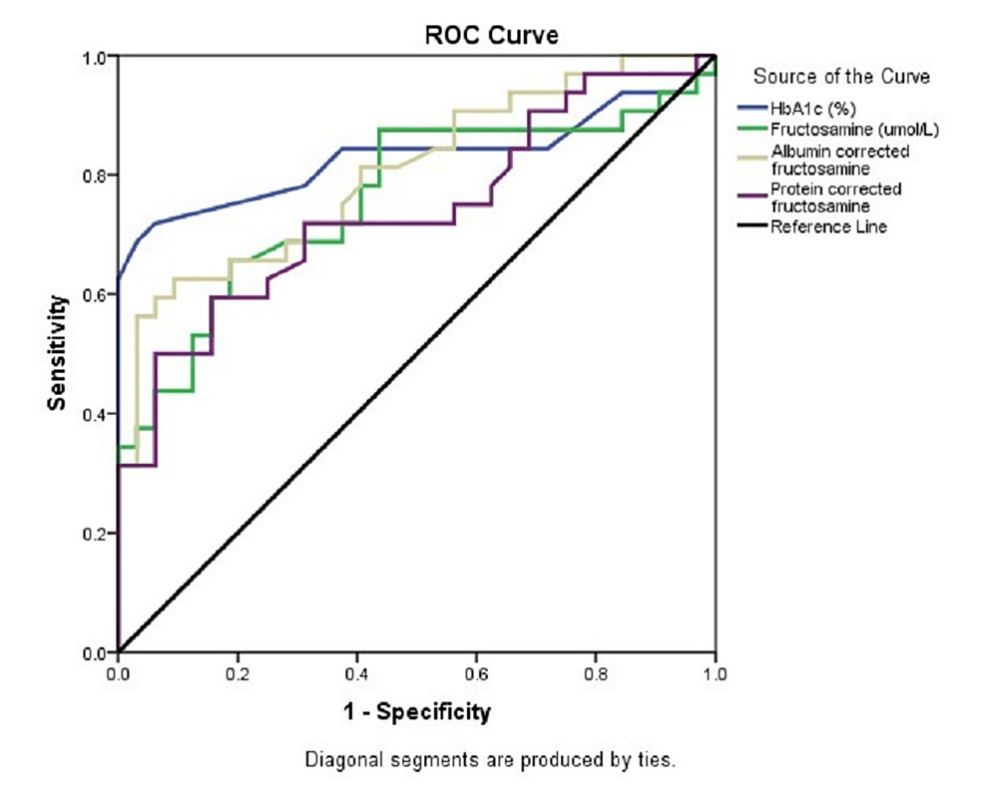
The ROC curve analysis for diagnostic efficiency of various parameters to detect diabetes mellitus HbA1c: Hemoglobin A1c, ROC: Receiver operating characteristic

The ROC curve analysis for assessing the efficiency of various parameters to detect DM was performed. Serum HbA1c was found to be the best parameter (AUC =83%, p<0.0001) followed by AlbF (AUC= 80.5%, p<0.0001) and uncorrected FA (AUC=80.5%, p<0.0001) (Table 5, Figure 10).

**Table 5:** The ROC curve analysis for diagnostic efficiency of various parameters HbA1c: Hemoglobin A1c, FA: Fructosamine, AlbF: Albumin-corrected fructosamine, PrF: Protein-corrected fructosamine, ROC: Receiver operating characteristic

Test Result Variables	Area	Std. Error	Asymptotic Significance	Asymptotic 95% Confidence Interval		Cut-off	Sensitivity	Specificity
				Lower Bound	Upper Bound			
HbA1c (%)	0.830	0.057	<0.0001	0.718	0.942	6.5	62.2	100
FA (umol/L)	0.760	0.062	<0.0001	0.638	0.881	443	65.6	81.3
AlbF	0.805	0.054	<0.0001	0.699	0.912	416	62.5	90.6
PrF	0.739	0.063	0.001	0.616	0.862	390	59.4	84.4

**Figure 11:**
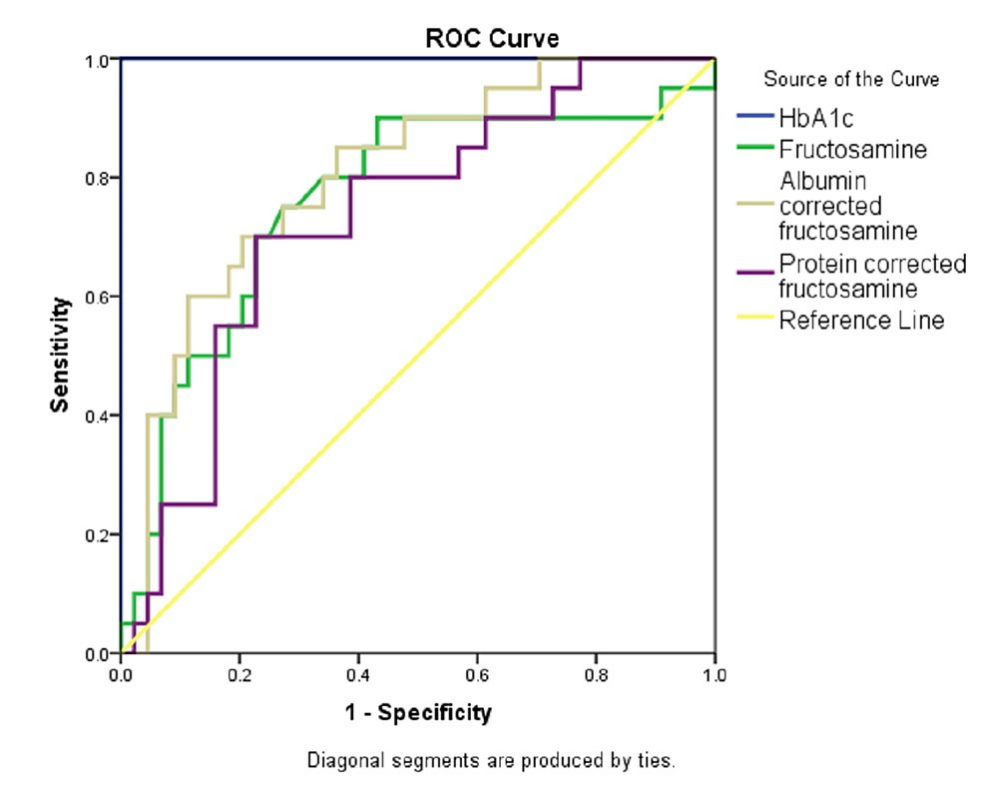
The ROC curve analysis of various parameters to detect diabetic range hyperglycemia ROC: Receiver operating characteristic, HbA1c: Hemoglobin A1c

The ROC curve analysis of various parameters to detect diabetic range hyperglycemia was performed. Serum HbA1c was found to be the best parameter (AUC =100%, p<0.0001) followed by AlbF (AUC= 80.3%, p<0.0001) and uncorrected FA (AUC=76.2%, p<0.0001) (Table 6, Figure 11).

**Table 6:** The ROC curve analysis of various parameters to detect diabetic range hyperglycemia HbA1c: Hemoglobin A1c, FA: Fructosamine, AlbF: Albumin-corrected fructosamine, PrF: Protein-corrected fructosamine, ROC: Receiver operating characteristic

Test Result Variables	Area	Std. Error	Asymptotic Significance	Asymptotic 95% Confidence Interval		Cut-off	Sensitivity (%)	Specificity (%)
				Lower Bound	Upper Bound			
HbA1c (%)	1.000	0.000	<0.001	1.000	1.000	6.5	100	100
FA (umol/L)	0.762	0.070	0.001	0.625	0.899	443	75	72.7
AlbF	0.803	0.058	<0.001	0.690	0.917	416	70	79.5
PrF	0.732	0.066	0.003	0.602	0.862	390.82	70	77.3

## Discussion

The current strategies concerning diagnosis and/or prognosis of DM are restricted to measuring HbA1c or fasting blood glucose (FBG) levels [6]. The FBG levels are amenable to variations due to dietary factors, therapeutic agents as well as metabolic variations from person to person [11]. Serum HbA1c, which is otherwise considered a robust parameter to be used in diagnosis as well as monitoring of DM, sometimes falls short of utility due to patient-related factors such as interferences by hemoglobin variants, decreased red blood cell life span (such as those with hemoglobinopathies, hemolytic anemia, etc.), end-stage renal disease patients, iron deficiency anemia, or in genetically hyperglycated population. Imprecise estimations of HbA1c might result if non-HPLC methods not complying with International Federation of Clinical Chemistry (IFCC) standards are used. Also, HbA1c has cost constraints in practical use [11,12]. Thus, both these parameters, despite being routine parameters for the diagnosis and monitoring of diabetes mellitus, have their disadvantages.

Fructosamine has gained importance as a new biomarker for hyperglycemia in the recent past. Serum FA is synthesized by spontaneous non-enzymatic glycation of serum proteins, at the amino-terminal of lysine and arginine by the Maillard reaction. The Schiff base product thus formed as an aldimine intermediate, then undergoes an Amadori rearrangement to finally yield a ketoamine linkage product, the FA molecule. By the virtue of its abundance in plasma protein, FA is principally glycated albumin (GA) [13].

In the present study, the mean levels of serum HbA1c, FA, AlbF, as well as PrF, were found to be significantly higher in the cases as compared to the controls. Also, correlation analysis revealed a significantly positive correlation between serum FA levels and the glycation status of the patients as suggested by their HbA1c levels. Thus confirming the diagnostic utility of FA. No significant correlation between FA and its corrected values for albumin and total protein obtained overall or in any range of diabetic control suggests that FA levels may not be required to be corrected for albumin or total protein levels in diabetic patients without any known special conditions such as pregnancy, chronic kidney disease(CKD), hemodialysis, etc. Also, no significant correlation was obtained between HbA1c, FA corrected for albumin or total protein with various hemoglobin states, indicating that there is no significant effect of hemoglobin levels over glycated Hb or other parameters used for monitoring of long-term glycemic control in uncomplicated DM. A moderate correlation was obtained between HbA1c levels and FA (r=0.479, p=0.033), AlbF (r=0.444, p=0.050), and PrF (r=0.441, p=0.065) in subjects with diabetic range glycemia. However, no such correlation was noted in the pre-diabetic group, indicating a linear relationship of FA, PrF, and AlbF with HbA1c at the diabetic range HbA1c. The lack of correlation in the non-diabetic and pre-diabetic range HbA1c population may be attributed to the low sample size.

Serum HbA1c is the parameter of choice for monitoring glycemic control over a long duration (two to three months). However, many conditions warrant more dynamic monitoring of blood sugar control. Some of these conditions include patients experiencing rapid changes in glucose homeostasis or wider glycemic excursions, those with a decreased life span of red blood cells, chronic renal diseases, etc. [14]. By the virtue of the fact that the average life span of non-immunoglobulin serum proteins is approximately 14 to 21 days, the measurement of serum FA provides information with respect to glycaemic control over the previous two to three weeks [15]. Also, the rate of non-enzymatic glycation of albumin is approximately nine to 10-fold higher for FA than that of HbA1c [16]. In a cross-sectional and longitudinal study carried out by Malmström et al. in 10,987 subjects, serum FA was found to be effective in differentiating subjects with and without diabetes (AUC, 0.95), displaying 61% sensitivity and 97% specificity at a threshold level of 2.5 mmol/L [17]. In the present study, we found AlbF to be the second-best parameter in diagnostic efficiency followed by uncorrected FA on ROC curve analysis. This emphasizes that serum FA may be a useful and valid diagnostic biomarker and probably of indispensable use in some special populations where HbA1c falls short of utility. Also, by providing information on short-term glycemic control, it is expected to reflect poor glycemic control better when compared to HbA1c. It thus seems to be a promising biomarker for monitoring purposes in patients wherein rapid excursions in glucose levels are expected such as in patients on corticosteroid therapy, hemodialysis, or on insulin therapy, etc.

Serum FA alone, however, may not make an excellent candidate for monitoring blood sugar control as the rate of its formation and concentration is dependent on blood glucose as well as protein concentrations. It may thus get altered in conditions affecting protein metabolisms such as protein-losing enteropathy, nephrotic syndrome, pregnancy, malnutrition, and decreased synthesis of proteins such as in liver cirrhosis, etc. There still, therefore, remains an iota of uncertainty with regard to its clinical usefulness. This can be circumvented by correcting the FA levels for serum AlbF or serum PrF [18]. In the present study, we obtained a moderate correlation between HbA1c levels and AlbF and PrF levels in the diabetic range group. However, no such correlation was noted in the prediabetic group. Lee et al. in their study conducted on 25 diabetic patients undergoing peritoneal dialysis, obtained a significant correlation between the interstitial fluid glucose levels obtained by continuous blood glucose monitoring system (CGMS) vs FA (r = 0.45, p<0.05) and AlbF levels (r = 0.54, p<0.01) [19]. Mittman et al. attempted to compare HbA1c levels with serum FA in monitoring glycemic control and associated morbidities in diabetic patients in a large urban hemodialysis center [20]. They enrolled 100 diabetic hemodialysis patients in their study and followed them up prospectively for three years. They found out that HbA1c and AlbF levels were significantly correlated with serum glucose levels, especially AlbF with mean glucose values of <150 mg/dl, and that it was a more clinically useful predictor of morbidity and hospitalization [20]. Similarly, Zhou et al. in their study with 2,238 elderly subjects aged 80 years and older, studied the associations between FA, AlbF, fasting plasma glucose (FPG), and mortality in the diabetic and non-diabetic subpopulations, and compared which marker better predicts mortality among the participants. They concluded that higher concentrations of FA, AlbF, and FPG were associated with a higher risk of all-cause or non-CVD mortality in the older population and that AlbF is a good glycemic predictor of mortality. Serum FA, therefore, has a potential role not only in the diagnosis and monitoring but also in the management of diabetic patients [18].

There are, however, a large number of clinical trials carried out in patients with renal failure which report not-so-significant correlations between serum FA levels and glycemic control [21,22]. These studies have inferred that FA is not a reliable marker for short to moderate duration of cumulative blood glucose index in diabetic patients with CKD. In the study conducted by Joy et al. with 23 diabetic hemodialysis patients, it was found that FA was not significantly associated with long-term glycemic control in diabetic patients receiving hemodialysis (r=0.345, p=0.11) [23].

For the pregnant population, HbA1c is known to exhibit a characteristic pattern of decrease in HbA1c values in the first and second trimesters owing to the unstable and overall decrease in blood glucose levels and an increasing trend in the third trimester which can be attributed to decreased blood relative iron deficiency secondary to haemodilution [24]. In another study by Khan et al. wherein fasting blood glucose and serum FA were estimated in 165 pregnant subjects, it was reported that serum FA can help to identify high-risk pregnant women prone to gestational DM, thus skipping the unnecessary glucose challenges required [25]. However, in contradiction to these findings, Li et al. measured FA in 161 pregnant women and inferred that serum FA is not a very good candidate for predicting gestational DM in early pregnancy due to the poor correlations with the outcome of the OGTT. However, they reported that this biomarker may be useful for identifying patients at higher risk of abnormal glucose tolerance [26]. Further large-scale prospective studies need to be carried out in this regard to establish the utility of serum FA in special populations such as those undergoing hemodialysis, pregnancy, etc. Special consideration may be given to analyzing AlbF or PrF in such cases.

The strengths of the present study are a well-characterized (as per ADA diagnostic criteria) and sub-classified cases group. Many studies carried out on studying glycemic control in diabetic patients have observed the diabetic group as a whole. To the best of our knowledge, the present study is the first of its kind to study the correlation of serum FA levels in diabetic groups sub-classified into pre-diabetes and frank diabetes. Also, the geographical location of our study which lies in Central India is a well-known high-prevalence belt for hemoglobinopathies such as sickle cell disease and thalassemia. This study may thus prove to be a pilot study for future large-scale prospective studies for other regions of India or the African subcontinent where a large subset of the population suffers from various hemoglobinopathies, thereby throwing more light on the clinical utility of FA. We have also tried to analyze FA corrected for albumin and total protein levels as they may seem to confound the results, especially in cases where protein metabolisms may be deranged. The limitation of this study was a smaller sample size. Further large-scale prospective studies need to be carried out in this regard to establish the findings of this study.

## Conclusions

The present study establishes its uniqueness in that the patient groups were classified into diabetic and pre-diabetic groups to study the correlation of HbA1c with FA levels. Also, the effect of hemoglobin levels on HbA1c and FA levels was assessed in patients with uncomplicated DM. Corrections for albumin and total protein levels were also carried out to understand the effectiveness of their correction required in special cases.

In the present study, the mean levels of serum FA, AlbF, as well as PrF, were found to be significantly higher in the cases as compared to the controls, as well as correlation analysis revealed a significantly positive correlation between serum FA levels and the glycation status of the patients as suggested by their HbA1c levels. Also, on ROC curve analysis, AlbF was found to be the second-best parameter in diagnostic efficiency followed by uncorrected FA. Thus, serum FA should be considered a valid diagnostic biomarker and probably of indispensable use in special populations where HbA1c falls short of utility such as in patients with red blood cell disorders or those showing rapid glycemic excursions such as on corticosteroid therapy or insulin therapy, etc. Its other advantages of being a faster and simpler technique, which can easily be automated with micro-sample volumes on many conventional laboratory instruments based on simple colorimetry along with low reagent costs, makes it a better choice for easy accessibility in centers where HPLC-based sophisticated instrumentation is difficult. Thus, it can especially be of help to low-income countries to better tackle the pandemic of diabetes.
